# A Nanoparticle Comprising the Receptor-Binding Domains of Norovirus and *Plasmodium* as a Combination Vaccine Candidate

**DOI:** 10.3390/vaccines13010034

**Published:** 2025-01-01

**Authors:** Ming Xia, Pengwei Huang, Frank S. Vago, Wen Jiang, Xi Jiang, Ming Tan

**Affiliations:** 1 Division of Infectious Diseases, Cincinnati Children’s Hospital Medical Center, 3333 Burnet Avenue, Cincinnati, OH 45229, USA; ming.xia@cchmc.org (M.X.); pengwei.huang@cchmc.org (P.H.); 2 Department of Biological Sciences, Purdue University, West Lafayette, IN 47907, USA; fvago@purdue.edu (F.S.V.); jiang12@purdue.edu (W.J.); 3 Department of Pediatrics, University of Cincinnati College of Medicine, Cincinnati, OH 45229, USA

**Keywords:** norovirus, *Plasmodium*, norovirus P particle, nanoparticle, αTSR domain, nanoparticle vaccine

## Abstract

Background: Noroviruses, which cause epidemic acute gastroenteritis, and *Plasmodium* parasites, which lead to malaria, are two infectious pathogens that pose threats to public health. The protruding (P) domain of norovirus VP1 and the αTSR domain of the circumsporozoite protein (CSP) of *Plasmodium* sporozoite are the glycan receptor-binding domains of the two pathogens for host cell attachment, making them excellent targets for vaccine development. Modified norovirus P domains self-assemble into a 24-meric octahedral P nanoparticle (P_24_ NP). Methods: We generated a unique P_24_-αTSR NP by inserting the αTSR domain into a surface loop of the P domain. The P-αTSR fusion proteins were produced in the *Escherichia coli* expression system and the fusion protein self-assembled into the P_24_-αTSR NP. Results: The formation of the P_24_-αTSR NP was demonstrated through gel filtration, electron microscopy, and dynamic light scattering. A 3D structural model of the P_24_-αTSR NP was constructed, using the known cryo-EM structure of the previously developed P_24_ NP and P_24_-VP8* NP as templates. Each P_24_-αTSR NP consists of a P_24_ NP core, with 24 surface-exposed αTSR domains that have retained their general conformations and binding function to heparan sulfate proteoglycans. The P_24_-αTSR NP is immunogenic, eliciting strong antibody responses in mice toward both the norovirus P domain and the αTSR domain of *Plasmodium* CSP. Notably, sera from mice immunized with the P_24_-αTSR NP bound strongly to *Plasmodium* sporozoites and blocked norovirus VLP attachment to their glycan receptors. Conclusion: These data suggest that the P_24_-αTSR NP may serve as a combination vaccine against both norovirus and *Plasmodium* parasites.

## 1. Introduction

Noroviruses belong to the genus *Norovirus*, in the family *Caliciviridae*. They are non-enveloped, single-stranded, positive-sense RNA viruses and are a leading cause of acute gastroenteritis (AGE), affecting individuals of all ages, in both developed and developing countries. These viruses spread rapidly, causing large AGE outbreaks in settings such as schools, cruise ships, military installations, and hospitals [1]. The global burden of norovirus-associated diarrhea is particularly severe in low- and middle-income countries, where it accounts for over 200,000 deaths annually, with USD 4.2 billion in direct health system costs and USD 60.3 billion in socioeconomic losses [2,3]. In the United States alone, norovirus infections cause an estimated 20 million cases of AGE each year, resulting in approximately 70,000 hospitalizations and up to 800 deaths [4,5]. Consequently, noroviruses remain a significant threat to global public health.

Each norovirus virion is encapsulated by an icosahedral capsid composed of capsid proteins (VP1s). The capsid comprises two major parts: an inner shell formed by shell (S) domains and multiple surface protrusions formed by the protruding (P) domains of VP1 [6]. The icosahedral inner shell is responsible for the structural integrity of the virus [7], while the surface protrusions play critical roles in the host interaction and immune response [8]. Accordingly, the S domains are genetically conserved, whereas the P domain sequences vary among different norovirus strains. However, receptor-binding sites within the P domain remain conserved within a genogroup, as these are crucial for receptor binding and viral survival [9,10]. Therefore, the P domain represents an excellent target for vaccine development. Previous studies have demonstrated that in vitro expression of norovirus P domains resulted in the self-assembly of dimers [11,12,13,14,15,16] and 24-meric P_24_ nanoparticles (P_24_-NPs) [17,18] through homotypic interactions. The P_24_ NP has been proposed as a norovirus vaccine candidate and as a platform for displaying antigens from other pathogens to develop combination vaccine candidates (reviewed in [19]).

*Plasmodium* parasites, a group of unicellular protozoans, are causative agents, causing malaria, a serious and sometimes fatal illness [20]. According to the World Health Organization (WHO), there was an estimated 241 million clinical cases of malaria globally in 2020, where approximately 627,000 of those cases resulted in death [21], underscoring malaria as a significant public health threat. Malaria is a mosquito-borne disease, transmitted when infected mosquitoes inject *Plasmodium* sporozoites into human skin through their bites [22]. The sporozoites migrate to the liver, where they replicate in hepatocytes and form merozoites [23,24]. These merozoites subsequently infect erythrocytes, proliferating further and causing the symptoms of malaria sickness [25,26]. Some merozoites also develop into gametocytes [27,28].

The sporozoites introduced by mosquito bites are exposed to host antibodies during their migration to the liver [20,29], making them a prime target for prophylactic and therapeutic strategies to prevent malaria. These sporozoites are enveloped by a multifunctional protein known as the circumsporozoite protein (CSP) [30]. Importantly, the CSP enables sporozoites to recognize heparan sulfate proteoglycans (HSPGs) on liver cells, facilitating their invasion of the liver [30,31,32,33]. Our prior study identified the αTSR domain (region II plus) [34] of the CSP as the HSPG binding domain [35], suggesting that the αTSR domain is an ideal target for inhibiting CSP function.

Vaccination is a highly effective prophylactic strategy for preventing infectious diseases. Currently, there are no commercially available vaccines for norovirus, although several candidates have progressed to clinical trials [36,37,38]. These include recombinant virus-like particle (VLP)-based vaccines administered via intramuscular injection [39,40,41,42,43] and an adenovirus-vectored VP1 protein-based vaccine for oral delivery [44]. In contrast, two malaria vaccines have been licensed for public use. The first one is Mosquirix^TM^, also known as RTS,S/AS01 [45,46,47,48,49], which incorporates the C-terminus and central repeats of the CSP from *Plasmodium falciparum*, displayed on recombinant hepatitis B virus core particles at a 20% ratio. The second is R21/Matrix-MTM [50,51,52,53,54,55], which also targets the CSP, but is displayed on recombinant hepatitis B virus core particles at a 100% ratio. Both vaccines require three to four doses to achieve a modest protective efficacy, providing approximately 75% protection for up to three years. Given these limitations, there is an urgent need for an effective norovirus vaccine and next-generation malaria vaccines with enhanced efficacy.

In this study, we developed a unique P_24_-αTSR NP incorporating the receptor-binding domains from both norovirus and *Plasmodium* parasites. Evidence supporting its potential as a combination vaccine candidate against these two pathogens includes the robust antibody responses elicited by the P_24_-αTSR NP in mice against both the norovirus P domain and the αTSR domain of *Plasmodium* CSP. Additionally, sera from mice immunized with the P_24_-αTSR NP specifically bound to CSPs on *Plasmodium* sporozoites and blocked norovirus VLP attachment to glycan receptors.

## 2. Materials and Methods

### 2.1. DNA Constructs for the Expression of P-αTSR Fusion Proteins

**Figure 1:**
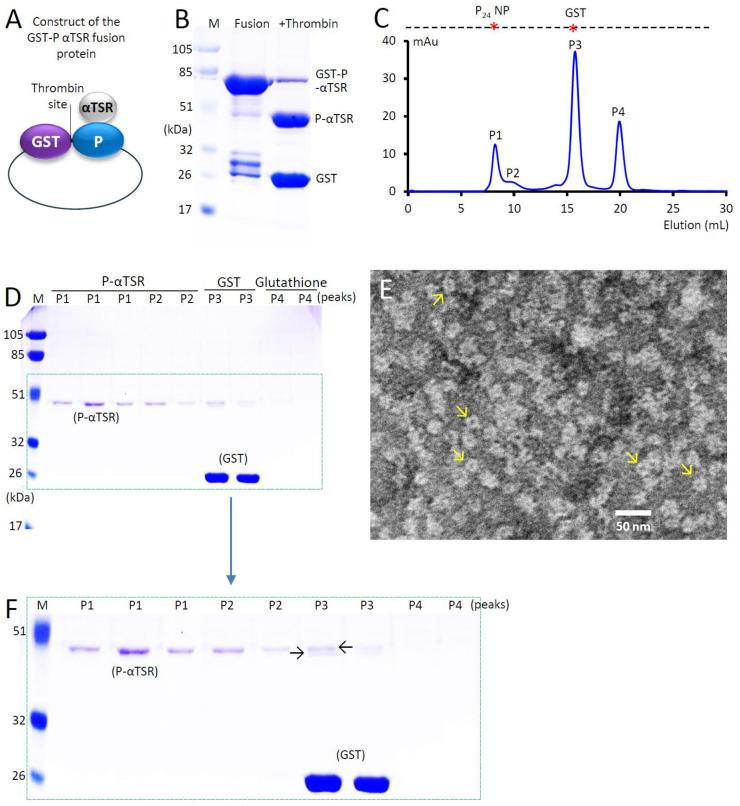
Production of the GST-tagged P-αTSR protein and self-formation of the P_24_-αTSR NPs. (**A**) Schematic representation of the GST-tagged P-αTSR fusion protein construct. The αTSR domain is inserted in loop 2 of the norovirus P domain, with a thrombin cleavage site present between the GST and P-αTSR protein. (**B**) SDS-PAGE analysis of the GST-P-αTSR fusion protein and its bipartite components after thrombin cleavage. Left lane (M): prestained protein standards, with molecular weights (MWs) indicated in kDa. Middle lane (Fusion): resin-purified GST-P-αTSR fusion protein (~68 kDa) with minor co-purified proteins. Right lane (+Thrombin): two separate components, the P-αTSR protein (~42 kDa) and the GST (~26 kDa), along with minor amounts of uncleaved GST-P-αTSR fusion protein, after thrombin cleavage. (**C**) Elution curve from gel-filtration chromatography of the thrombin-cleaved GST-P-αTSR protein, showing four distinct elution peaks (P1, P2, P3, and P4). The *Y*-axis represents relative protein amounts measured by ultraviolet absorbance at 280 nm (mAU) and the *X*-axis indicates the elution volume (mL). The dashed line at the top, marked with two red stars, indicates the elution positions of the P_24_ NP (MW ~830 kDa) and GST dimer (MW~52 kDa). (**D**) SDS-PAGE analysis of the four peaks from the gel-filtration chromatography, showing the presence of P-αTSR and/or GST protein in each peak. (**E**) A representative transmission electron microscopy (TEM) image of the P-αTSR protein from P1 in (**C**,**D**), showing the formation of the P_24_-αTSR NPs. Arrows point to typical P_24_-αTSR NPs. (**F**) Enlarged SDS-PAGE image in (**D**), highlighting the double bands of the P-αTSR protein from P3 in (**C**), indicated by arrows.

**Figure 2:**
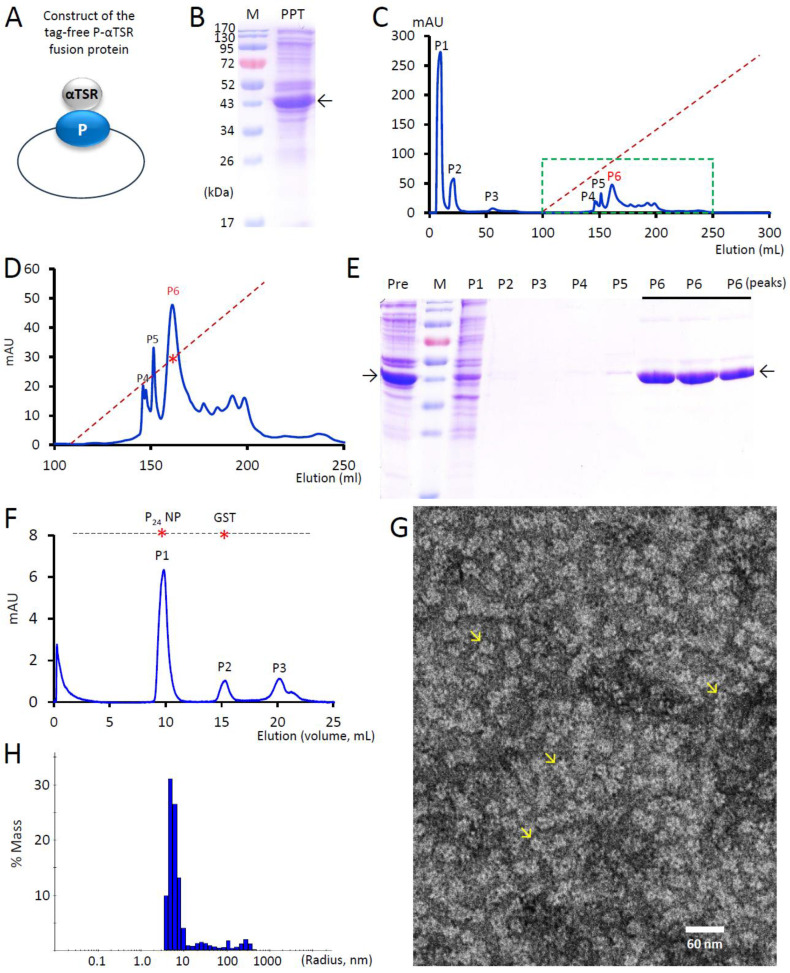
Generation and characterization of tag-free P-αTSR protein and P_24_-αTSR NPs. (**A**) Schematic representation of the tag-free P-αTSR protein construct. (**B**) SDS-PAGE analysis of the P-αTSR protein precipitated with ammonium sulfate [(NH4)_2_SO_4_]. The left lane (M) shows prestained protein standards, with molecular weights (MWs) labeled in kDa. The right lane (PPT) displays the P-αTSR protein (denoted by an arrow) precipitated from bacterial lysate using 1.2 M (NH4)_2_SO_4_. (**C**,**D**) Representative elution profile from anion exchange chromatography of the (NH4)_2_SO_4_-precipitated P-αTSR protein. (**D**) Is an enlarged view of the framed region in (**C**). The *Y*-axis represents relative protein amounts in the eluents, measured by ultraviolet absorbances at 280 nm (A280, mAU), while the *X*-axis indicates the accumulated elusion volume (mL). The dashed red line shows the linear gradient of buffer B (0–100%), with a red star marking the percentages of buffer B at the elution peak of the P-αTSR protein (33.9%, P6). Six major elution peaks (P1 to P6), analyzed by SDS-PAGE in (**E**), are indicated. (**E**) SDS-PAGE analysis of the six major elution peaks from the anion exchange chromatography in (**C**,**D**). Lane “Pre”: redissolved ammonium sulfate-precipitated protein before loading. Lane M: prestained protein standards with MWs indicated in kDa, as in (**B**). The tag-free P-αTSR protein (~68 kDa) is present in P6, as indicated by an arrow. (**F**) Elution curve from gel filtration of the purified tag-free P-αTSR protein. The curve shows a single major peak in the void volume (P1), corresponding to the P_24_-αTSR NP (MW > 800 kDa). Two minor peaks (P2 and P3) appeared. P2 likely represents dimeric P-αTSR protein with an MW similar to the GST dimer (~52 kDa), while P3 may represent degraded protein fragments with MWs < 10 kDa. The *Y*-axis indicates relative protein amounts, while the *X*-axis represents the elution volume, as in (**C**,**D**). The dashed line at the top, marked with two stars, indicates the elution positions of the P_24_ NP (~830 kDa) and GST dimer (~52 kDa). (**G**) Representative transmission electron microscopy (TEM) image of the P-αTSR protein from P1 in (**F**), showing the formation of the P_24_-αTSR NPs. Arrows point to typical P_24_-αTSR NPs. (**H**) The particle size distribution of the tag-free P_24_-αTSR NPs, determined by dynamic light scattering (DLS).

The DNA fragment encoding the αTSR domain of the *P. falciparum* 3D7 strain (GenBank AC#: CAB38998.2, from E309 to C375, 67 amino acids) was amplified using PCR from a previously constructed plasmid containing the αTSR-encoding sequence [35]. This fragment was then subcloned into surface loop 2 of the norovirus P domain in a separate, previously created pGEX-4T-1-based plasmid encoding the glutathione S-transferase (GST)-P-VP8* fusion protein [56], replacing the VP8*-encoding sequence. The resulting plasmid was used to produce GST-tagged P-αTSR fusion protein (Figure 1A), with a thrombin cleavage site between the GST and the P-αTSR protein. An additional plasmid was constructed by subcloning the P-αTSR-encoding DNA sequence into the pET-24b vector (Figure 2A) to produce the tag-free P-αTSR protein, with a stop codon introduced before the C-terminal Hisx6-encoding sequence in the vector.

### 2.2. Generation of the P-αTSR Proteins

The P-αTSR proteins were produced in *Escherichia coli* BL21 Arctic strain, through induction with 0.25 mM isopropyl-β-D-thiogalactopyranoside (IPTG) at 13 °C for 16 h, as described elsewhere [13,17]. Soluble GST-tagged P-αTSR protein was isolated from the bacterial lysate using GST-binding resin (Glutathione Sepharose 4 Fast Flow, GE Healthcare). Following thrombin cleavage, the GST tag was removed from the P-αTSR protein via gel-filtration chromatography (see below). Additionally, soluble tag-free P-αTSR protein, expressed via the same *E. coli* system, was precipitated from the bacterial lysate with 1.2 M ammonium sulfate [(NH_4_)_2_SO_4_] and further purified using anion exchange chromatography (see below).

### 2.3. SDS-PAGE Analysis and Protein Quantitation

Recombinant proteins produced in this study were analyzed using sodium dodecyl sulfate polyacrylamide gel electrophoresis (SDS-PAGE), with 12% separating gels. The protein concentrations were quantified either by comparison to known concentrations of bovine serum albumin (BSA, Bio-Rad, Hercules, CA, USA) on the same gels [56] and/or by using a NanoDrop spectrophotometer.

### 2.4. Gel-Filtration Chromatography

This method was carried out using an ÄKTA Fast Performance Liquid Chromatography system (FPLC, ÄKTA pure^TM^ 25 L, GE Healthcare Life Sciences, San Francisco, CA, USA), with a size-exclusion column (Superdex 200, 10/300 GL, 25 mL bed volume, GE Healthcare Life Sciences), as described previously [13]. The elution peaks corresponding to the P_24_-αTSR NPs and GST were identified using the previously prepared norovirus P_24_ NPs (~830 kDa) [18] and GST dimers (~52 kDa) [35]. The relative protein amount in the eluent was monitored by its absorbance at 280 nm (A_280_).

### 2.5. Anion Exchange Chromatography

This procedure was performed using the same ӒKTA FPLC system (see above), equipped with a HiPrep Q HP 16/10 column (GE Healthcare Life Sciences), as previously described [57,58]. Briefly, the column was equilibrated with seven column volumes (CVs) of 20 mM tris buffer (pH 8.0, buffer A). After the protein sample was loaded, unbound proteins were washed out using seven CVs of buffer A. The bound proteins, including the target protein, were eluted using a linear gradient (0 to 100% B) over eight CVs of 1 M NaCl in buffer A (buffer B). The column was subsequently washed using seven CVs of buffer B, followed by re-equilibration with seven CVs of buffer A. The relative protein concentrations in the eluent were monitored by UV absorbances at 280 nm (A_280_, mAU). The elution positions of the target proteins are indicated as percentages of buffer B.

### 2.6. Cesium Chloride (CsCl) Density Gradient Ultracentrifugation

This approach was used to assess the density of the P_24_-αTSR NPs, following a previously established protocol [59]. A 0.5 mL sample of purified tag-free P-αTSR protein was mixed with 10 mL of a CsCl solution, at a density of 1.3630 g/mL. The mixture was centrifuged at 288,000× *g* for 45 h, using an Optima L-90K ultracentrifuge (Beckman Coulter, Brea, CA, USA). The resulting gradient was fractionated into 23 fractions using a bottom puncture. Each fraction was diluted 100-fold with PBS, coated onto 96-well microtiter plates, and analyzed for the presence of the P-αTSR protein using a guinea pig hyperimmune serum against the norovirus VLP [60] and mouse hyperimmune serum against the αTSR protein. The CsCl densities of the fractions containing the P-αTSR protein were determined using the refractive index method.

### 2.7. Transmission Electron Microscopy (TEM)

Negative stain TEM was used to visually examine the morphology of the P-αTSR NPs, following a previously described protocol [59]. Briefly, purified P-αTSR fusion proteins were absorbed onto grids (FCF200-CV-50, Electron Microscopy Sciences, Hatfield, PA, USA) and stained with 1% ammonium molybdate. The grids were air dried and examined using a Hitachi electron microscope (model H-7650) at 80 kV, with magnifications ranging from 15,000× to 40,000×.

### 2.8. P_24_-αTSR NP Glycan Binding Assay

This procedure was carried out as previously described [35]. Briefly, heparin sulfate (HS) in the form of heparin sodium salt purified from porcine intestinal mucosa (Sigma-Aldrich, St. Louis, MO, USA) at a concentration of 25 µg/mL was coated onto 96-well microtiter plates (Thermo Fisher Scientific, Waltham, MA, USA). The P_24_-αTSR NP, at different concentrations, was incubated with the coated heparin, with the GST-tagged αTSR [35] serving as a positive control and the P_24_ NP [18] and GST serving as negative controls. Bound P_24_-αTSR NP/P_24_ NP proteins were detected using guinea pig hyperimmune serum against norovirus VLP [60] at 1:3000 dilution, while bound GST-αTSR/GST proteins were detected using mouse hyperimmune serum against GST-αTSR [35] at 1:2000 dilution. Horseradish peroxidase (HRP)-conjugated goat anti-guinea pig IgG or mouse IgG (Thermo Fisher Scientific) at a 1:5000 dilution was used to measure the bound antibodies. Finally, HRP substrates were added to determine the signal intensity, which was defined by the optical density (OD) at 450 nm.

### 2.9. Dynamic Light Scattering (DLS)

A total of 200 µL of P-αTSR proteins was placed into a well of a clear flat-bottom 96-well microplate (Greiner Bio-One, Monroe, NC, USA) and analyzed with the DynaPro Plate Reader III DLS instrument (Wyatt Technology, Goleta, CA, USA). The size distribution of the P-αTSR NPs was determined using DYNAMICS software (Wyatt Technology).

### 2.10. Structural Modeling of the P_24_-αTSR NP

A 3D structural model of the P_24_-αTSR NP was constructed using UCSF ChimeraX software (version 1.4) [61], based on cryo-EM (cryogenic electron microscopy) density maps of the norovirus P_24_ [18] and the P_24_-VP8* NPs [56] as templates. In this model, the VP8* domains were replaced with the crystal structures of the αTSR domains of *P. falciparum* (PDB code: 3VDJ) [34]. UCSF ChimeraX software was also utilized for the structural analysis of the P_24_-αTSR NP model and for generating visualizations.

### 2.11. Immunization of Mice

A total of 24 pathogen-free BALB/c mice, at an age of ~8 weeks, were randomly divided into three groups of 8 mice each (n = 8). Each group was immunized intramuscularly (IM) three times at 2-week intervals with one of following three immunogens at a dose of 10 µg/mouse/dose: (1) P_24_-αTSR NP; (2) Hisx6-tagged αTSR protein [35]; and (3) P_24_ NP [18]. All immunogens were administered with aluminum salt adjuvant (Imject Alum, Thermo Fisher Scientific), at a dose of 25 μL/mouse/dose (20 μg/mouse/dose). Sera samples were prepared from blood specimens that were collected two weeks after the third immunization via cardiac puncture.

### 2.12. Specific IgG Titer Determination

Serum IgG antibody titers specific to *Plasmodium* αTSR and the norovirus P domain were determined using enzyme immunoassays (EIAs). Briefly, purified GST-αTSR [35] or P_24_ protein at 5 µg/mL were used as capture antigens and coated onto 96-well microtiter plates. The coated antigens were blocked with 5% non-fat milk and then hatched with diluted mouse sera. IgG that bound the coated antigens was detected by HRP-conjugated goat-anti-mouse IgG (1:5000, MP Biomedicals, Irvine, CA, USA). The αTSR and P domain-specific IgG titers were defined as the highest serum dilutions yielding positive signals (OD450 ≥ 0.2).

### 2.13. Blocking of Norovirus VLP–Glycan Receptor Interaction

The norovirus P domain binds to host cell surface histo-blood group antigens (HBGAs) to initiate infection. This binding has been mimicked in an established EIA-based binding assay, with serum antibodies that block this interaction considered surrogate neutralizing antibodies against norovirus [60]. The blocking assay begins by coating HBGAs, in the form of a well-defined type A saliva sample, onto 96-well microtiter plates. Norovirus GII.4 VLPs [60] were pre-incubated with serially diluted sera obtained from mice immunized with the P_24_-αTSR NPs or controls and then added to the wells with coated HBGAs. A reduction in the binding signal, compared to wells without serum blocking, indicates the blocking effect.

### 2.14. Immunofluorescence Assays (IFAs)

Sera from mice immunized with the P_24_-αTSR NP or the P_24_ NP control were used to stain the circumsporozoite proteins (CSPs) on the surface of *P. falciparum* sporozoites, following a procedure reported previously [62,63]. Briefly, slides with air-dried sporozoites of *P. falciparum*, kindly provided by Dr. Photini Sinnis at Johns Hopkins University, were brought to room temperature and blocked with 1% BSA in 1× Tris-Buffered Saline (TBS, pH 7.4). In a humidity chamber, mouse sera diluted at 1:8000 were incubated with the sporozoites. After washing, the sporozoites were incubated with a fluorophore-conjugated secondary antibody (Millipore-Sigma, Norwood, OH, USA), mounted with Citifluor Mountant Media, and sealed with a cover glass using nail polish. The sporozoites were observed with a fluorescence microscope at 20× to 40× magnifications.

### 2.15. Ethics Statement

The animal study in this project was carried out in accordance with the recommendations outlined in the Guide for the Care and Use of Laboratory Animals (23a) by the National Institute of Health (NIH). The study procedures were reviewed and approved by the Institutional Animal Care and Use Committee (IACUC) at Cincinnati Children’s Hospital Research Foundation (Animal Welfare Assurance No. A3108-01).

### 2.16. Statistical Evaluation

Statistical comparisons between the data groups were conducted using GraphPad Prism 9.0 (GraphPad Software, Inc. Boston, MA, USA) and an unpaired *t* test. Differences were deemed not significant at *p*-values > 0.05, significant at *p*-values < 0.05 (marked as “*”), significant at *p*-values < 0.01 (marked as “**”), and highly significant at *p*-values < 0.001 (marked as “***”).

## 3. Results

### 3.1. Production of the P-αTSR Fusion Protein

The P-αTSR protein was initially generated as a soluble GST-tagged protein (Figure 1A) via an *E. coli* expression system, yielding approximately 20 mg of target protein per liter of bacterial culture. This was achieved by isolating the target protein from the bacterial lysate using GST-binding resin (Figure 1B, middle lane, ~68 kDa). The purified GST-P-αTSR fusion protein was then cleaved using thrombin, resulting in two proteins: a ~42 kDa P-αTSR protein and a ~26 kDa GST (Figure 1B, right lane). The two proteins were separated by gel-filtration chromatography, followed by SDS-PAGE analysis (Figure 1C,D). Gel filtration revealed three major peaks, corresponding to the P-αTSR protein in the form of P_24_-αTSR NPs (P1, see below), GST (P3), and glutathione (P4) from the elution buffer, respectively.

The P-αTSR protein was also produced using a tag-free approach (Figure 2). After expression in the *E. coli* system, the P-αTSR protein was precipitated from the bacterial lysate using 1.2 M ammonium sulfate (Figure 2B). The precipitated P-αTSR protein, along with co-precipitated bacterial proteins, was dissolved in 20 mM tris buffer (pH 8.0) and subjected to anion exchange chromatography, resulting in multiple peaks (Figure 2C–E). The P-αTSR protein was eluted in P6, corresponding to 33.9% of buffer B (339 mM NaCl, Figure 2C–E), with a yield of approximately 15 mg of target protein per liter of bacterial culture.

### 3.2. Self-Assembly of the P_24_-αTSR NPs

During the gel-filtration chromatography used to separate the GST tag from the P-αTSR protein (Figure 1C), the majority of intact P-αTSR protein eluted as a peak (P1) in the void volume, indicating a molecular weight (MW) greater than the maximum mass (800 kDa) of the Superdex 200 size-exclusion column. This aligns with the calculated MW of 1008 kDa for the P_24_-αTSR NP (24 × 42 kDa), suggesting that the P-αTSR protein self-assembled into P_24_-αTSR NP, consistent with the known self-assembly propensity of the P domain protein into the P_24_ NP [17]. TEM analysis of the P-αTSR protein from the P1 elution revealed a particle morphology characteristic of P_24_ NPs, with a size of approximately 15 nm, showing some variations (Figure 1E), further confirming the formation of the P_24_-αTSR NPs. While the majority of the intact P-αTSR protein self-assembled into the P_24_-αTSR NPs, SDS-PAGE analysis (Figure 1D) identified some degraded P-αTSR proteins in P3, co-eluting with the GST dimers. The degraded P-αTSR proteins appeared as two bands, with the lower band indicating partial degradation, which may have impaired their ability to form P_24_-αTSR NPs (see Section 4).

Additional evidence supporting the self-formation of P_24_-αTSR NPs was obtained from the study of the P-αTSR protein purified using the tag-free approach. First, gel-filtration analysis of the purified P-αTSR protein from the P6 elution using the same Superdex 200 column revealed a major peak at the void volume (Figure 2F). Second, TEM inspection of the same protein sample showed particles with typical P_24_ NP morphology [18], with an approximate diameter of 15 nm (Figure 2G). Third, DSL analysis revealed that the purified P-αTSR protein formed NPs of diverse sizes, with the major population ranging in diameter from 10 to 20 nm (Figure 2H). Moreover, three minor peaks were observed, corresponding to particle sizes of 50, 200, and 500 nm. Collectively, these data confirmed that the purified P-αTSR protein self-assembled into the P_24_-αTSR NPs.

### 3.3. D Structural Modeling of the P_24_-αTSR NP

**Figure 3:**
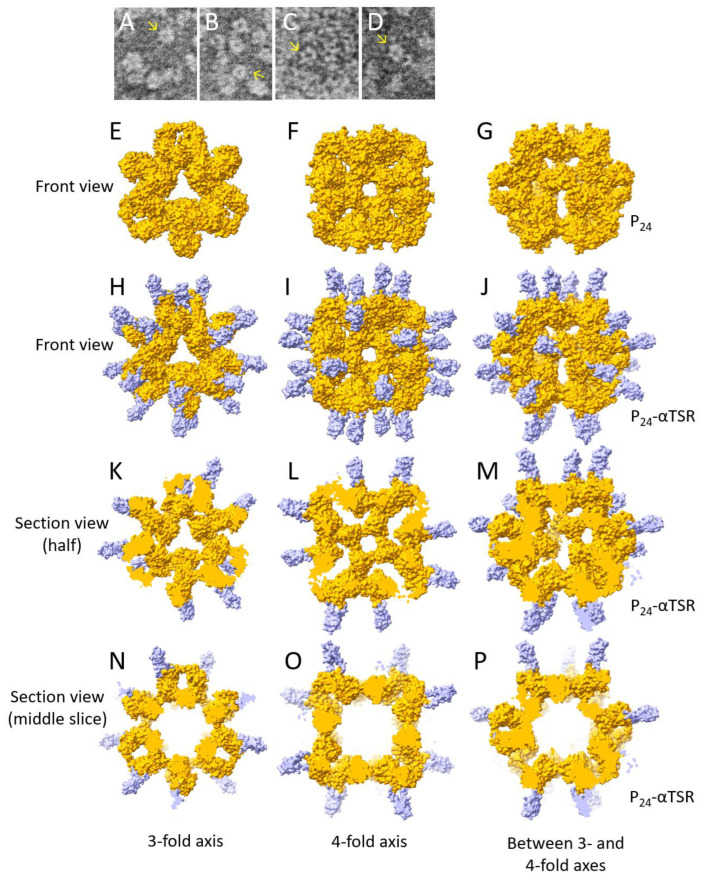
The 3D structural models of the P_24_-αTSR NP. The model was constructed using UCSF ChimeraX software version 1.4, based on the previously elucidated cryo-EM maps of the norovirus P_24_ NP and the P_24_-VP8* chimeric NP. In this model, the rotavirus VP8* domain was replaced by the known crystal structure of the αTSR domain (PDB code: 3VDJ). (**A**–**D**) Transmission electron microscopy (TEM) images showing the typical morphology of the P_24_-αTSR NP (indicated by arrows), consisting of a P_24_ NP core with multiple extended protrusions formed by the αTSR domains. (**E**–**G**) Surface representations of the norovirus P_24_ NP, viewed along the three-fold axis (**E**), the four-fold axis (**F**), and at an intermediate angle between these two axes (**G**). (**H**–**J**) Surface representations of the P_24_-αTSR NP, viewed along the three-fold axis (**H**), the four-fold axis (**I**), and at an intermediate angle between these two axes (**J**). (**K**–**M**) Cross-sectional views of the three P_24_-αTSR NP structures shown in (**H**–**J**), cut in half and viewed from the cutting planes along the same three symmetry axes as in (**H**–**J**). (**N**–**P**) Middle-section cross-sectional views of the three P_24_-αTSR NP structures shown in (**H**–**J**), viewed from the same three symmetry axes as in (**H**–**J**). In all images from (**H**) to (**P**), the P_24_ NP core is depicted in orange, while the αTSR domains are shown in purple.

TEM micrographs of the P_24_-αTSR NPs revealed the characteristic morphologies of the octahedral P_24_ NP [18], with recognizable extended protrusions corresponding to the αTSR domains (Figure 3A–D). Additional shapes were also observed in the micrographs. To better understand these morphologies, a 3D model of the P_24_-αTSR NP was constructed, using the cryo-EM structures of the P_24_ NP [18] (Figure 3E–G) and the P_24_-VP8* [56] NP as templates. This was achieved by fitting the known crystal structures of the αTSR domains of *P. falciparum* (PDB code: 3VDJ) [34] into the VP8* density maps of the P_24_-VP8* NP, using UCSF ChimeraX software. Based on this octahedral model, we generated representative images of the P_24_-αTSR NP from various viewing angles (Figure 3H–P), including views along the three-fold (Figure 3H,K,N) and four-fold (Figure 3I,L,O) symmetry axes, as well as intermediate angles between the two (Figure 3J,M,P). In summary, the octahedral P_24_-αTSR NPs do not display typical spherical shapes; instead, they may appear as rectangles, pentagons, and/or hexagons, depending on the viewing angle. These visualizations provide valuable insights into the diverse morphologies observed in the TEM micrographs.

### 3.4. Further Characteristics of the P_24_-αTSR NP

**Figure 4:**
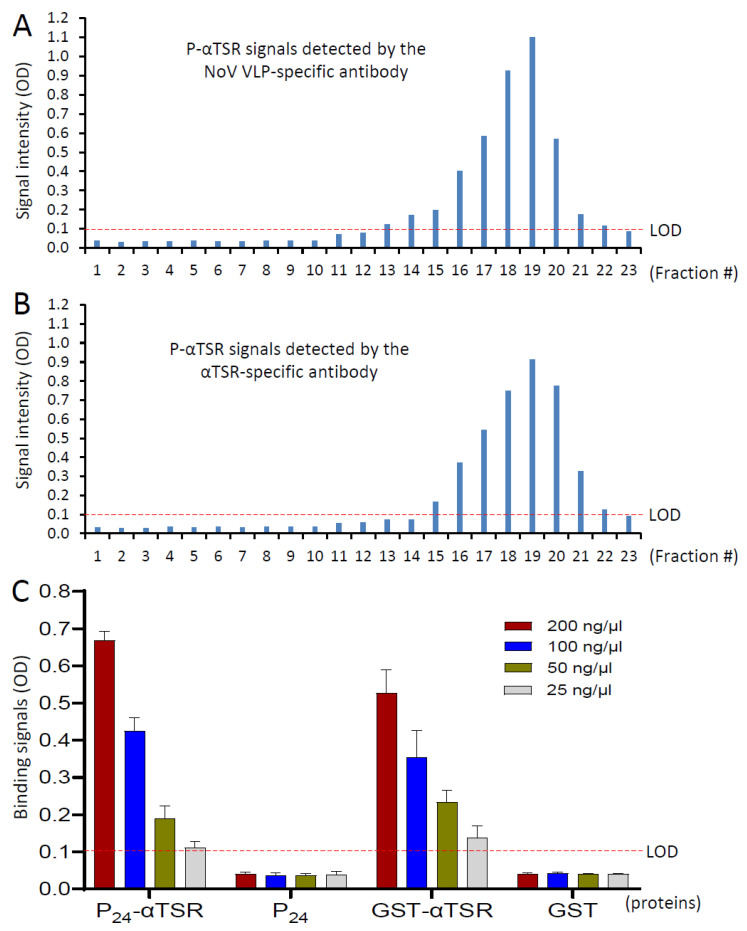
CsCl density gradient ultracentrifugation, specific antibody reactivity, and heparin sulfate glycan binding of the P_24_-αTSR NP. (**A**,**B**) Each of the 23 fractions from the CsCl density gradient was analyzed for the presence of the P_24_-αTSR NP using EIA assays. Hyperimmune serum from a guinea pig after immunization with norovirus VLP (**A**) and hyperimmune serum from a mouse after immunization with the *Plasmodium* αTSR domain (**B**) were used as detection antibodies, respectively. The *Y*-axis represents the optical density (OD) as a measure of signal intensity, while the *X*-axis corresponds to the gradient fractions arranged from the bottom (fraction 1) to the top (fraction 23). (**C**) EIA-based binding assay showing the interaction of the P_24_-αTSR NP with heparin sulfate glycans. The GST-αTSR protein served as a positive control, while the P24 NP and GST were used as negative controls. The *Y*-axis indicates the binding signal intensity of the OD, while the *X*-axis shows the different proteins at the indicated concentrations. The limit of detection (LOD), indicated by a red dashed line in each panel, was set at OD = 0.1.

The P_24_-αTSR NP was analyzed using a CsCl density gradient. Following ultracentrifugation, the gradient was fractionated, followed by detection of the P_24_-αTSR NPs in the fractions through EIAs using hyperimmune sera against norovirus VLP [60] and αTSR [35]. This revealed an overlapped peak in the upper half of the gradient, centered at fraction 19 (Figure 4A,B), with a density of 1.2995 g/cm^3^. These results confirmed the bipartite composition of the P_24_-αTSR NP, consisting of the norovirus P_24_ NP core and the *P. falciparum* αTSR domains, which form the surface protrusions (Figure 3). Furthermore, the specific recognition of the P_24_-αTSR NP by targeted antibodies validated the preservation of the native conformations of both the norovirus P domain and the αTSR domain within the NP.

### 3.5. Interaction of the P_24_-αTSR NP with Heparin Glycans

The αTSR domain has been shown to interact with HSPGs [35]. We demonstrated that the P_24_-αTSR NP, with its surface-exposed αTSR domains, interacted with heparin sulfate in a dose-dependent manner, similar to the GST-αTSR protein (Figure 4C). In contrast, the P_24_ NP and GST, used as negative controls, did not exhibit such interactions. These findings validated that the αTSR domains displayed on the P_24_ NP retain their glycan receptor-binding function, further supporting the conclusion that αTSR domains maintain their native conformations when displayed on the P_24_ NP.

### 3.6. Robust Antibody Response to the Bipartite Composition of the P_24_-αTSR NP

**Figure 5:**
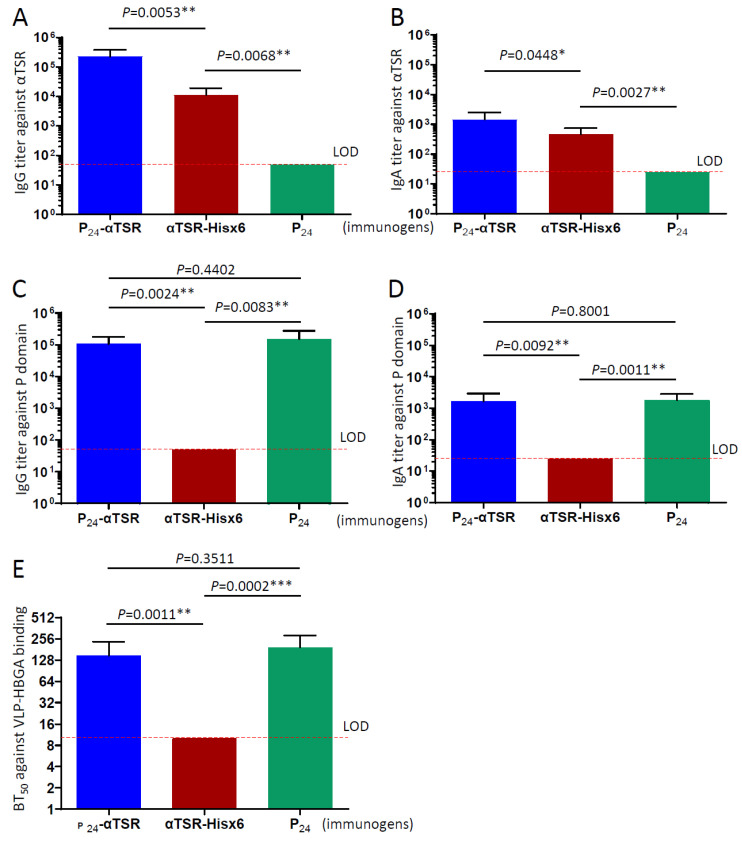
Immune responses to the P_24_-αTSR NP toward its bipartite components. (**A**,**B**) *Plasmodium* αTSR-specific IgG (**A**) and IgA (**B**) titers in mice immunized with the P_24_-αTSR NP (blue column) compared to titers induced by free αTSR (red column) and the P_24_ NP (green column) after three intramuscular immunizations. (**C**,**D**) Norovirus P domain-specific IgG (**C**) and IgA (**D**) titers in mice immunized with the P_24_-αTSR NP (blue column) compared to titers induced by free αTSR (red column) and the P_24_ NP (green column) after three intramuscular immunizations. In (**A**,**C**), the Y-axes represent αTSR-specific (**A**) or P domain-specific (**C**) IgG titers. In (**B**,**D**), the Y-axes represent αTSR-specific (**B**) or P domain-specific (**D**) IgA titers. In (**A**–**D**), the X-axes indicate various immunogens. (**E**) The 50% blocking titers (BT50) of sera from mice immunized with the P_24_-αTSR NP (blue column), P_24_ NP (green column), and free αTSR (red column) against norovirus VLP-glycan receptor attachment. The *Y*-axis represents BT50 values, while the *X*-axis indicates various immunogens. Corresponding statistical *p* values between the data groups are displayed above the columns. The limit of detection (LOD) is indicated by a red dashed line in each panel. Differences were deemed not significant at *p*-values > 0.05, significant at *p*-values < 0.05 (marked as “*”), significant at *p*-values < 0.01 (marked as “**”), and highly significant at *p*-values < 0.001 (marked as “***”).

Serum IgG and IgA responses specific to the norovirus P domain and *P. falciparum* αTSR domain were evaluated in mice following immunization with the P_24_-αTSR NP (Figure 5A–D). The norovirus P_24_ NP and free αTSR (with His tag), prepared previously [18,35], were included as controls. After three immunizations, the P_24_-αTSR NP induced a significantly higher αTSR-specific IgG titer of 1:226,575, compared to the titer of 1:12,200 elicited by the free αTSR protein (*p* = 0.0053, Figure 5A). Conversely, the P_24_-αTSR NP elicited a high P domain-specific IgG titer of 1:106,057, comparable to the titer of 1:149,943 induced by the P_24_ NP (*p* = 0.4402, Figure 5C).

IgA responses to the two components of the P_24_-αTSR NP followed similar trends, but were lower in magnitude than the IgG responses (Figure 5B,D). Specifically, the P_24_-αTSR NP elicited a significantly higher αTSR-specific IgA titer of 1:1466, compared to 1:467 induced by the free αTSR protein (*p* = 0.0448, Figure 5B). However, the P_24_-αTSR NP induced a P domain-specific IgA titer of 1:1667, which was comparable to the titer of 1:1828 elicited by the P_24_ NP (*p* = 0.8001, Figure 5D). As expected, the norovirus P_24_ NP did not induce *Plasmodium* αTSR-specific IgG or IgA responses (Figure 5A,B) and the free αTSR protein did not elicit norovirus P domain-specific antibodies (Figure 5C,D).

### 3.7. Inhibition Against Norovirus VLP–Glycan Receptor Interactions

Norovirus–HBGA attachment is a critical initial step in norovirus infection [64], which can be assessed using an EIA-based binding assay [60]. Studies using clinical trial samples have established a correlation between serum blockade of the norovirus VLP–HBGA interaction and protection against norovirus infection and disease [65,66,67]. Consequently, blockade of the norovirus VLP–HBGA interaction is regarded as a surrogate marker for neutralization, with the serum functional antibody response assessed through the norovirus VLP–HBGA attachment blocking assay. We observed that sera from mice immunized with the P_24_-αTSR NP effectively blocked norovirus VLP–HBGA attachment, with a 50% blocking titer of 1:145.6, comparable to the titer of 1:194.3 observed in sera from mice immunized with P_24_ NP (*p* = 0.3511, Figure 5E). In contrast, sera from mice immunized with the αTSR protein did not exhibit such blocking effect. These findings suggest that the sera from mice immunized with the P_24_-αTSR NP possess the potential to neutralize norovirus infection and/or disease.

### 3.8. Staining of Plasmodium Sporozoites Using Mouse Hyperimmune Sera

**Figure 6:**
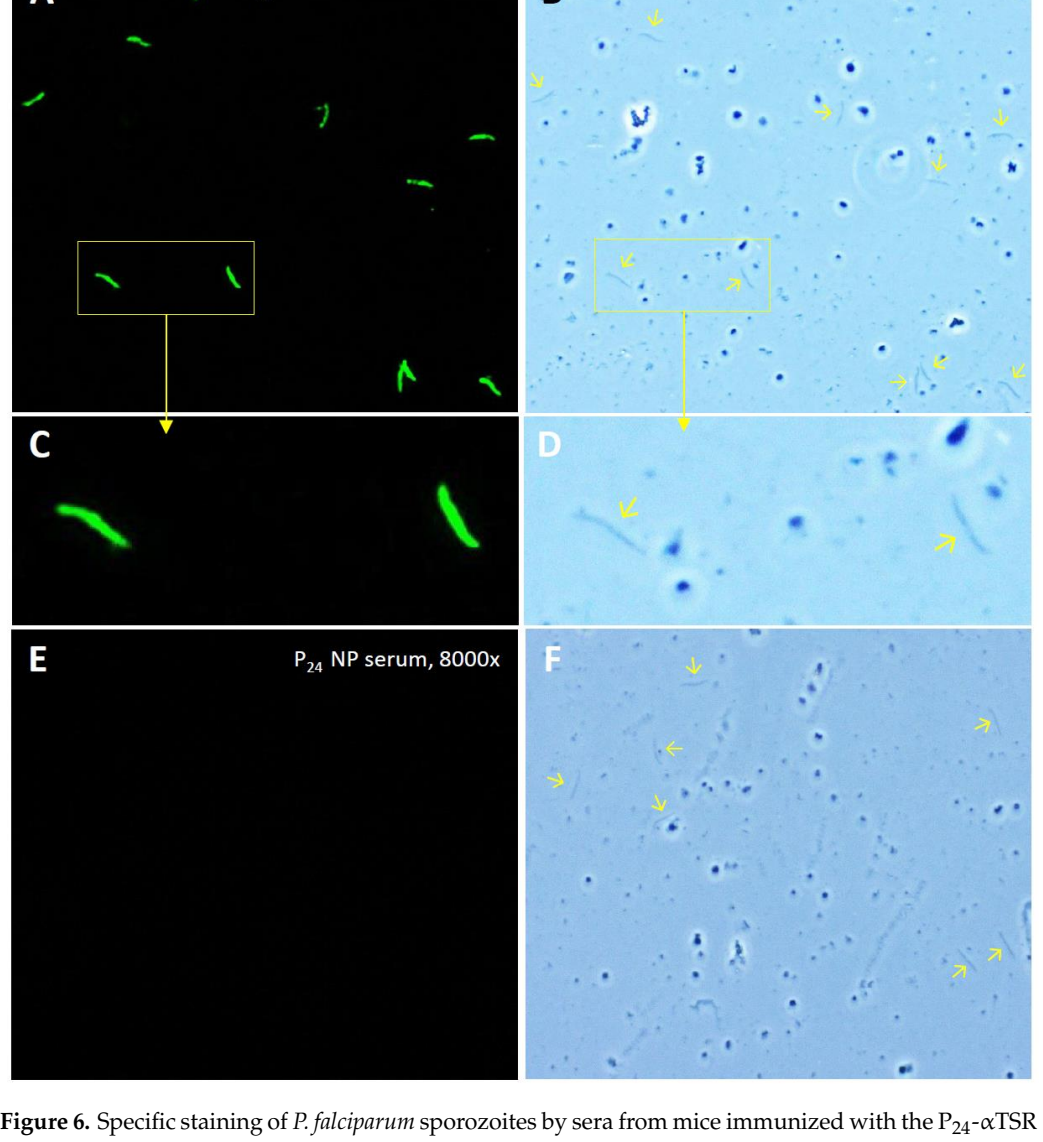
Specific staining of *P. falciparum* sporozoites by sera from mice immunized with the P_24_-αTSR NP, in immunofluorescence assays (IFAs). (**A**) Representative IFA micrograph showing *Plasmodium* sporozoites stained by mouse sera (1:8000 dilution) after three immunizations with the P_24_-αTSR NP. (**C**) Enlarged view of the marked regions in (**A**). (**E**) Sera (1:8000 dilution) from mice immunized with the P_24_ NP showed no staining of sporozoites (negative control). (**B**,**D**,**F**) Optical views of the same fields correspond to the IFA images in the left panels. Arrows indicate the visualized sporozoites.

The sera from mice immunized with the P_24_-αTSR NP were evaluated for their binding capability in regard to surface-exposed CSPs on *Plasmodium* sporozoites, using an IFA method. Two mouse sera with average αTSR-specific IgG titers were tested, and representative micrographs are shown in Figure 6. The air-dried *P. falciparum* sporozoites on slides were specifically stained by the sera from mice immunized with the P_24_-αTSR NP at 8000-fold dilution (Figure 6A–D), whereas the control sera from mice immunized with the P_24_ NP at the same dilution did not produce staining (Figure 6E,F). The staining was highly specific, as non-sporozoite materials surrounding the sporozoites (Figure 6B,D) remained unstained, whereas the sporozoite surface was evenly stained by the sera. Further dilutions of the sera were not performed due to the limited availability of air-dried *P. falciparum* sporozoites on the slides. The observed specific binding of the hyperimmune sera to CSPs on the sporozoites strongly suggests that the αTSR-specific antibodies may inhibit the function of the αTSR domain of CSPs, potentially blocking the binding of *Plasmodium* sporozoites to their glycan receptors. Thus, the P_24_-αTSR NP shows potential as a vaccine candidate against *Plasmodium* sporozoite infection and malaria.

## 4. Discussion

In this study, we leveraged the self-assembly properties of the P_24_ NP, derived from the modified norovirus protruding (P) domain through homotypic interactions, to design and evaluate a novel chimeric NP. This NP, named P_24_-αTSR NP, displays the receptor biding αTSR domain of the *Plasmodium* parasite, with the ultimate goal of developing a combination vaccine targeting both pathogens and their associated diseases. The P_24_-αTSR NP was produced by inserting the *Plasmodium* αTSR domain within an exposed surface loop of the norovirus P domain, followed by the generation of the P-αTSR fusion protein through an *E. coli* expression system, using scalable methods. A series of experiments validated the self-formation of the P_24_-αTSR NP, consisting of a P_24_ NP core, with multiple αTSR domains forming the extended protrusions on its surface. Supporting evidence includes data from gel-filtration chromatography, a TEM inspection, DLS analysis, and structural modeling of the P_24_-αTSR NP.

Since both the norovirus P domain and the *Plasmodium* αTSR region are responsible for the attachment to the host glycan receptor, a critical step in initiating pathogenic infection, we further investigated the potential of the P_24_-αTSR NP as a dual vaccine candidate. Our results demonstrated that the P_24_-αTSR NP is highly immunogenic in mice, eliciting robust antibody responses against both the norovirus P domain and *Plasmodium* αTSR. Moreover, we showed that the serum antibodies obtained after immunization with the P_24_-αTSR NP blocked norovirus VLPs from attaching to their histo-blood group antigen (HBGA) receptors, an assay widely regarded as a surrogate for norovirus neutralization. Additionally, the serum antibodies bound specifically to circumsporozoite proteins (CSPs) on the surface of *Plasmodium* sporozoites. We hypothesize that this antibody–αTSR interaction may block the glycan receptor binding site of the αTSR in CSPs, thereby preventing the parasite from attaching to host receptors and, ultimately, inhibiting infection. Collectively, these findings support the potential of the P_24_-αTSR NP as a promising vaccine candidate against both norovirus and *Plasmodium* parasites, as well as the diseases they cause.

We observed some variability in the size, structure, and morphology of the P_24_-αTSR NPs in this study compared to similar NPs reported previously. For example, the morphologies of the P_24_-αTSR NPs observed through TEM appeared less homogeneous than those of the P_24_ NP described earlier [17]. This suggests that the insertion of *Plasmodium* αTSR domains into the surface loops on the protrusions of the P_24_ NPs may influence the global structures and morphologies of the resulting P_24_-αTSR NPs to some extent. One possibility is the formation of smaller NPs, referred to as P_12_-αTSR NPs, which comprise 12 norovirus P domains with a calculated MW of 504 kDa, similar to the P_12_ NP reported previously [68].

This hypothesis is supported by the presence of a minor elution peak (P2) in the gel-filtration chromatography, corresponding to a MW smaller than the major P_24_-αTSR NP elution peak (P1), observed during the analysis of the thrombin-cleaved GST-P-αTSR protein (Figure 1C,D). Additional evidence includes the observation of smaller NPs in the TEM micrographs that differ from the typical P_24_-αTSR NPs. Notably, the minor peak corresponding to the P_12_-αTSR NPs did not appear in the P-αTSR protein generated using the tag-free approach (compare Figure 1C with Figure 2F). Consistently, TEM inspection revealed better homogeneity in regard to the size and morphologies of the NPs produced via the tag-free method (compare Figure 1E with Figure 2G). These findings suggest that the tag-free procedure is a superior approach for producing P_24_-αTSR NPs compared to the GST fusion method. Moreover, the tag-free procedure is easier to scale up as it eliminates the protease cleavage step, which may degrade the target protein to some extent. In fact, such degradation was observed, as evidenced by the appearance of double protein bands representing the P-αTSR protein after thrombin cleavage (Figure 1C,D). These degraded P-αTSR proteins seem to hinder their assembly into P_24_-αTSR NPs.

The diverse morphologies of the octahedral P_24_-αTSR NPs observed in the TEM micrographs in this study may be attributed to variations in viewing angles or symmetry axes, as illustrated by the 3D structural model (Figure 3). It is important to emphasize that, although some P_12_-αTSR NPs may be present among the predominant P_24_-αTSR NPs, their negative impact on the immune response outcomes is likely minimal. This is because both NP forms share the primary positive factors influencing the immune response of an antigen: (1) Both NP forms appear to preserve authentic pathogen-associated molecular patterns (PAMPs) in the bipartite components, as demonstrated by their strong reactivity to antibodies against norovirus VLP and the *Plasmodium* αTSR domain. (2) Both NPs have repetitive antigen patterns, promoting multiple stimulations of the host immune system. (3) Both NPs have a large molecular size, serving as intrinsic adjuvants to enhance the immune response. The observed robust antibody responses, high amount of blocking antibody titers against norovirus VLP–glycan receptor attachment, and strong binding ability to *Plasmodium* sporozoites support this hypothesis. Finally, DLS showed that the majority the P-αTSR NP sizes ranged from 10 to 20 nm, consistent with the sizes observed in the TEM. However, DLS also revealed three minor NP populations, corresponding to particle sizes of 50, 200, and 500 nm. These much larger NP sizes may be attributed to aggregation of the P-αTSR protein or the P-αTSR NPs.

A limitation of this study is our inability to evaluate the cell culture-based neutralization or animal model-based protective efficacy of our vaccine candidate against both pathogens. This is due to the lack of a conventional cell culture system for human norovirus and the absence of a small animal model capable of mimicking norovirus infection and disease. However, since human norovirus can replicate to a certain extent in an enteroid culture system, we will collaborate with partners in the future to further assess the neutralization of our vaccine candidate using this platform. Furthermore, our laboratory currently lacks the capability to evaluate the protective efficacy of malaria vaccines. To address this, we plan to engage with potential collaborators who have the necessary expertise to assess the protective efficacy of our P_24_-αTSR NP vaccine candidate against *Plasmodium* infection.

Although *Plasmodium* parasite-associated malaria and norovirus-induced diarrhea are very different diseases, they are likely to affect similar populations. For example, malaria is known to be prevalent in tropical and subtropical areas, particularly in Africa [69]. Similarly, global surveillance data shown that Africa has the highest prevalence of norovirus infection, with a rate of 15% [70]. Therefore, populations in Africa may be susceptible to both pathogens, justifying the usefulness of a combination vaccine against the two diseases.

## 5. Conclusions

In this study, we developed and evaluated a novel P_24_-αTSR NP, which incorporates the receptor-binding domains of both norovirus and *Plasmodium* parasites. Our findings demonstrate its potential as a dual vaccine candidate against these two pathogens. This is evidenced by the robust antibody responses it elicited against the norovirus P domain and the αTSR domain of *Plasmodium* CSP. Moreover, sera from immunized mice bound specifically to CSPs on *Plasmodium* sporozoites and inhibited the attachment of norovirus VLPs to glycan receptors.

## Data Availability

The data presented in this study are available in this article.
